# The impact of digital economy development on public health: evidence from Chinese cities

**DOI:** 10.3389/fpubh.2024.1347572

**Published:** 2024-07-12

**Authors:** Hao Li, Yu Li

**Affiliations:** ^1^School of Business, Xinyang Normal University, Xinyang, China; ^2^Research Institute of the Economic and Social Development in the Dabie Mountains, Xinyang, China

**Keywords:** digital economy, public health, comprehensive health, heterogeneity analysis, transmission path

## Abstract

**Introduction:**

The swift advancement of the digital economy not only plays a crucial role in stimulating a country’s economic growth but also exerts a significant influence on national health and well-being.

**Methods:**

Adopts the data of 285 prefecture-level cities in China from 2010 to 2022, and employs the panel data fixed-effects model, spatial Durbin model, and mediated effects model to study the impact of digital economic development on the level of urban public health.

**Results:**

It is found that digital economic development significantly improves the level of urban public health, and the effect has a spatial spillover effect. The impact of digital economic development on urban public health is mainly concentrated in cities with higher levels of economic development, higher levels of digital economic development, and lower levels of urban public health. Among them, technological innovation and information dissemination are the main dissemination channels through which digital economic development affects the level of urban public health.

**Discussion:**

The advancement of the digital economy significantly impacts urban public health. It is advisable to bolster policies guiding digital economy development, enhance cross-sector collaboration between the digital economy and public health, reinforce public health education and awareness campaigns, improve the digital health literacy of the population, continue to enhance the fairness and accessibility of basic medical services, and address and bridge the “digital divide” between regions, as well as urban and rural areas.

## 1 Introduction

Since the dawn of the 21st century, the digital economy has emerged as the most rapidly growing and influential new economic model within China’s pursuit of high-quality economic development. The digital economy’s empowering effects and ecological features have become pivotal in reorganizing global resources, reshaping the world’s economic structure, and altering the landscape of global competition. Additionally, the real-time, interactive, and multimedia capabilities of digital technology have seamlessly woven into the fabric of everyday life, significantly impacting the physical and mental well-being of individuals worldwide. In the 3 years following the COVID-19 outbreak, China has employed digital tools like location and travel codes in its response to COVID-19. By utilizing these tools, authorities have effectively tracked citizens’ movements. Through digital nucleic acid testing, they have categorized the risk levels of the epidemic, allowing for precise interventions in crucial locations, among specific demographics, and in critical situations. This strategy of integrating digital technology with health protection ensures traceability, predictability, and quantifiability in the management of COVID-19, bolstering the efficacy of epidemic risk control. It furnishes crucial insights for government entities to make precise decisions in prevention and control efforts, safeguarding individuals’ lives and health to the utmost extent. The forthcoming 5 years are poised to be pivotal for initiating the comprehensive development of a socialist modern nation. Effectively driving the convergence of the digital economy with the medical and healthcare sector stands out as a critical imperative. This alignment aims to leverage China’s digital prowess to bolster public health outcomes, fostering both material prosperity and well-being. By enhancing the synergy between digital technologies and healthcare, the goal is to meet the evolving demands of the populace for an enhanced standard of living. Such efforts hold immense practical significance in expediting the creation of a more beautiful and healthy China.

People’s health serves as the cornerstone for the advancement of societal civilization and stands as a crucial indicator of national prosperity and well-being. In the 21st century, alongside the rise of the digital economy and the promotion of a people-centric health ethos, the intersection of digital economic progress with public health has increasingly drawn scholarly attention. Existing research has delved into the influence of digital advancements, including Internet proliferation and mobile phone utilization, on the mental health, lifestyle choices, and physical fitness attitudes across various age demographics (1–3). There is a scarcity of literature that delves into the trajectory of digital technology within the healthcare sector, particularly concerning data security dilemmas. Additionally, there’s a lack of discussion on the nuanced variations and the underlying mechanisms of its effects. The significant question of delineating the direct influence of digital economy development on public health merits empirical investigation. In response, this study investigates the repercussions of digital economy growth on urban public health and its transmission mechanisms. In view of this, this article explores the effect of digital economy development on urban public health and its transmission paths by measuring the level of digital economy development and the comprehensive level of urban public health in 285 prefecture-level cities in China, setting up an econometric model and adopting various empirical methods. Firstly, this study broadens the scope from a singular dimension of Internet usage to encompass the multifaceted aspects of digital economy development, with a particular focus on its influence on urban public health. Secondly, by evaluating the degree of digital economy advancement and the overarching condition of urban public health, this paper thoroughly investigates the impact of the digital economy on urban public health. It employs methods such as the instrumental variable approach and the substitute variable technique to minimally reduce the model’s endogeneity issue, thereby enhancing the regression results’ robustness and securing more credible and applicable conclusions. Lastly, the study probes into the diversity of effects that digital economy development has on urban public health by considering factors such as urban geographical location, economic progress level, digital economy growth stage, and overall public health status. It also delves into the mechanisms through which digital economy advancement influences the level of urban public health. This research carries substantial theoretical and practical implications, aiming to foster digital economy growth and contribute to the endeavor of building a healthier China.

## 2 Mechanistic analysis and research hypothesis

There is a lack of academic consensus regarding the definition and core attributes of the digital economy. Typically, the term “digital economy” pertains to economic activities conducted over the Internet (4). In a narrow context, the digital economy includes transactions facilitated by electronic intermediary platforms and their associated entities. On a broader scale, the digital economy encompasses a wider range of digital economic activities (5). In the context of this study, the digital economy is defined as the application of digital technologies and associated economic endeavors leveraging the Internet, big data, artificial intelligence, communication technologies, among others (6).

The integration of digital technology into healthcare has paved the way for the dissemination of health-related knowledge and practices through various digital platforms. It entails the integration of health data, medical services, and health information through digital technology to enhance healthcare services, bolster health protection, and advance public health outcomes (7). This trend encompasses the utilization of digital technologies in health management, including the Internet of Things, Artificial Intelligence, and Big Data (8). The term “digital” signifies digital technologies such as the Internet, big data, and artificial intelligence, while “health” pertains to individuals in optimal physical, mental, and social well-being. Digital health involves the fusion of digital technology with healthcare, medicine, lifestyle, and society, with the objective of enhancing the effectiveness of medical services and facilitating the delivery of tailored and precise healthcare services (9). Drawing from established literature, the mechanisms through which the digital economy influences public health can be outlined as follows.

First, the technological impact of the digital economy extends the range of public health services and enhances the clinical treatment experience offered by healthcare institutions. This expansion encompasses Internet health information platforms, mobile digital health monitoring, online health status analysis, health consultation and advice services, big data diagnostics, intelligent telehealth devices, augmented reality, virtual reality, and other avenues for public involvement in healthcare (10). By shifting from closed monopolies to open information sharing, the digital economy facilitates the reorganization and enhancement of medical resources, enabling swift responses to diverse medical and health requirements (11). This approach enhances both traditional medical diagnostics and the overall healthcare service system, streamlining medical examinations, enhancing individual health risk management, and directly or indirectly bolstering urban public health (9). Consequently, this article posits Hypothesis 1.

*H1*: The digital economy enhances public health data mining and diagnostic capabilities through technological empowerment, thereby effectively advancing urban public health.

Second, the integration of technologies such as big data, cloud computing, and artificial intelligence with healthcare has spurred the development of innovations including family clinics, online healthcare services, private health assistants, and integrated solutions for health, retirement, tourism, fitness, and leisure, as well as food (7). This integration is also exploring advancements in wearable devices, intelligent health electronics, and healthcare mobile application services, thereby giving rise to new health industry sectors, business models, and operational frameworks (10). Offering the public services for healthy living, including health information access, fostering health awareness, managing health information, medical recreation, and health consultation, serves to advance personalized health management services (12). This initiative also nurtures distinct health management sectors tailored to individual needs. To this end, this article introduces Research Hypothesis 2.

*H2*: The evolution of the digital economy, underpinned by extensive public engagement, fosters the advancement of the healthcare industry, enhances the efficiency of the public health service continuum, and caters to the diverse health demands of the population.

Third, digital technology is poised to revolutionize healthcare by deeply integrating various societal stakeholders for collaborative governance and establishing a global system for sharing medical and health resources (13). This transformation addresses the comprehensive demand for public health management services, streamlines patients’ access and feedback mechanisms, and enhances the efficiency and oversight of governmental health regulation (11). Furthermore, it improves the effectiveness of digital and public health management, ensuring that medical services are universal, equitable, accessible, and affordable (14). By effectively narrowing the disparity in medical resources across different regions and between urban and rural areas, it helps to rectify the uneven distribution of healthcare resources (15). The advancement of the digital economy plays a crucial role in supporting governments to devise strategies for epidemic prevention and control, coordinate the allocation of healthcare resources, maintain health-related big data, mitigate health-related cognitive bias and anxiety, refine health decision-making processes, reform health governance models, and enhance the efficiency of health regulation (15). Consequently, the public health governance structure will become more streamlined and the governance process more transparent, facilitating real-time interconnection, data sharing, as well as collaborative efforts. This fosters precise regulation and scientific governance, propelling the modernization of the health governance system and enhancing its capacity (16). Therefore, we propose research hypothesis 3.

*H3*: The advancement of the digital economy enhances the efficiency of allocating medical and health resources, addresses challenges like unequal distribution of healthcare resources among regions, and bolsters the perception of accessibility and equity in public health services.

Fourth, the evolution of the digital economy, rooted in data-centric innovations, equips us with a comprehensive suite of capabilities for acquiring, utilizing, disseminating, sharing, and creating digital health content (9). This fosters a social health model predicated on online information dissemination. Such an approach assists the public in accessing and utilizing health information, maintaining and enhancing health awareness, developing and strengthening healthy behaviors, and optimizing personal lifestyles. It addresses the individualized needs of public health, aids in cultivating health literacy, and contributes to the formation of accurate health concepts among the public (1). The advancement of the digital economy not only offers the public a holistic view of health and diseases, empowering consumers to access more detailed and precise health information about themselves, but also presents new avenues and possibilities for governments to enhance health monitoring, disease control, and the efficiency of medical and healthcare services (9). Nevertheless, disparities in digital technology ownership, utilization, and innovation capabilities across regions and individuals can lead to information gaps and delays. This discrepancy can exacerbate issues of “wealth inequality” and the “digital divide” in technological access, impacting the enhancement of urban public health (15). Therefore, we propose research hypothesis 4.

*H4*: The advancement of the digital economy enhances public health by facilitating information dissemination, but its impact on urban public health varies due to the presence of a “digital divide” among different regions and demographic groups.

## 3 Research design

### 3.1 Model setting

Building on the health production function established by Grossman (17), this study incorporates variables such as the level of urban digital economic development, environmental influences, economic conditions, healthcare attributes, and public health factors into the framework to derive the following foundational model 1 (16, 18) Equation 1:


(1) $$\mathrm{He}l_{\mathrm{it}}=\alpha_{0}+\alpha_{1}Dig_{\mathrm{it}}+\sum \beta X_{\mathrm{it}}+\gamma_{i}+\lambda_{t}+\varepsilon_{\mathrm{it}}$$

Where, *Dig* represents the degree of digital economic advancement within the city, while *Hel* signifies the comprehensive level of urban public health. X encompasses a range of factors influencing public health levels. The coefficients $\alpha_{1},\beta$ correspond to the level of digital economic development and each respective control variable. γ accounts for regional-specific effects, while λ reflects temporal effects. ε represents the stochastic disturbance term in the model.

### 3.2 Variable selection

Explanatory variable: Comprehensive level of urban public health. Health is a complex and abstract notion intricately linked to individuals’ socio-cultural backgrounds, knowledge structures, religious convictions, ethical practices, humanistic attributes, and laws (19). Public health extends beyond the physical and mental well-being of the populace to encompass their capacity to adapt and thrive across physical, mental, and social dimensions, striving toward an optimal quality of life (3). Diverse perceptions of health among individuals lead to variations in the selection of health evaluation criteria, yielding disparate assessment outcomes. To address the limitations of conventional health metrics characterized by uni-dimensionality and partiality, researchers often develop comprehensive health index evaluation systems. For instance, the World Health Organization (WHO) formulates holistic health indicators across four dimensions: physical, mental, moral, and social well-being. In China, the Ministry of Health advocates for a health evaluation index system comprising physical status, healthcare facilities, and environmental and behavioral factors. Beyond fundamental health indicators, scholars also incorporate variables like environmental quality, lifestyle choices, social influences, environmental health, and healthcare provisions into health level assessments (20). Nevertheless, existing studies primarily focus on provincial-level comprehensive health indicator systems, neglecting the measurement and dynamic comparison of urban public health comprehensiveness (16, 20). Moreover, enhancing public health often hinges on healthcare security, restricted by local medical and public health service capabilities (21). Hence, developing a comprehensive health level evaluation system tailored to Chinese urban settings necessitates considering not only residents’ health foundations but also health security aspects linked to public medical services provision. To capture the essence of health and portray the actual health status of the populace, this paper constructs a health index with continuity, dynamism, and comparability through the lenses of “health foundation” and “health security,” prioritizing representativeness, scientific rigor, and reliability. See Table 1 for details.

**Table 1:** Indicator system for the level of urban public health.

Primary index	Secondary index	Indicator description	Unit	Index attribute
Health foundation	Life expectancy	Population health level and survival time	Year	+
	Mortality	Hygiene practices and quality of care	‰	−
	Proportion of moderate malnutrition in children under 5 years of age	Nutritional status and level of the population’s diet	%	−
	Incidence of Class A and B legally reported infectious diseases	Evolution of infectious disease development	1/100,000	−
Health security	Number of hospitals per million population	Medical institution supply, universal level	Individual	+
	Number of practicing physicians per million population	*Per capita* health personnel supply level	People	+
	Number of registered nurses per million population	Number of practicing physicians per million population	People	+
	Number of health technicians per million population	Number of practicing physicians per million population	People	+
	Number of hospital beds per 10,000 population	Scale of health hardware facilities	Zhang	+
	Medical institution bed utilization rate	Degree of utilization of medical and health resources	%	+
	Average hospital stay	Medical benefits and technology level	Heaven	−
	Average number of resident visits	Degree of utilization of medical and health resources	times	+
	Total government health investment costs as a percentage of GDP	Percentage of total government health spending	%	+

Due to the limitation of space, the measurement results are not shown in detail.

The primary explanatory variable is the level of digital economy development in cities. Domestic scholars typically consider various dimensions of the digital economy, such as digital infrastructure, value added in the digital industry, and output efficiency, when assessing the overall development of the digital economy in cities. This article, drawing insights from relevant sources, establishes an indicator system for measuring the level of city digital economy development. The system is based on the digital economy industry framework outlined by the (22) and consists of four dimensions: digital economy infrastructure, digital economy industrialization, industry digitization, and digital governance (23), as illustrated in Table 2.

**Table 2:** Indicator system for the level of urban digital economy development.

Primary index	Secondary index	Indicator description	Unit
Digital economy infrastructure	Internet penetration rate	Number of Internet users as a proportion of resident population	%
	Telephone penetration rate	Landline and mobile phones with Ratio of total population in administrative areas	Department
	Length of long distance fiber optic cable routes	Length of long distance fiber optic cable routes	10,000 km
	Internet broadband access port	Internet broadband access port	Million
	Number of Internet domain names	Number of Internet domain names	Million
Digital industrialization	Gross industrial output value of the digital industry	Communication equipment, computers and others total industrial output value of electronic equipment manufacturing	Billion
	Scale of electronic information manufacturing	Computers, communications and other number of listed companies in electronic equipment manufacturing	Individual
	Size of the software industry	Number of listed companies in the software and IT services sector	Individual
Digitalization of industry	Size of the radio and television industry	Number of listed companies in radio, television, film and film recording production	Individual
	Digital industry practitioners	Average year-end employees in the information transmission, software and information technology services industry	Number of persons
	Software business revenue	Software business revenue	Million
	Total telecommunications business	Total telecommunications business	Billion
	Number of digital TV subscribers	Number of digital TV subscribers	10,000 Households
	E-commerce scale	Number of listed companies in the e-commerce business	Individual
	Intelligent production	Number of listed companies in the intelligence business	Individual
	Enterprise information technology level	Proportion of companies adopting information management	%
	Digital inclusive finance index	Peking University Digital Inclusive Finance Index	
	Number of courier operations	Courier volume	10,000 pieces
Digital governance	Digital e-Government Level	Number of government websites	Individual
	Number of new government media	Official government accounts on new media platforms	Individual

Due to the limitation of space, the measurement results are not shown in detail.

The methodology for measuring the comprehensive index is detailed in this article. It focuses on 285 prefecture-level cities in China between 2010 and 2022, excluding cities with substantial missing data due to constraints on data availability. Leveraging panel data characteristics, the study uses 2010 as the baseline year. Initially, the original data is standardized through a polarization method, followed by the application of an enhanced longitudinal and horizontal pull-out method to assign weights to the fundamental indicators. Subsequently, a linear weighting method is employed to compute the comprehensive urban public health level and the index for digital economy development. The data sources selected for this study include variables from city statistical yearbooks, Health Yearbooks, the China Health Statistical Yearbook, the China Traditional Chinese Medicine Yearbook, among others. Some data are collected from local government websites and Internet health reports. To address missing data in certain years, the article utilizes a proximity mean approach for supplementation and enhancement.

Building on relevant research, this article incorporates the following control variables (1, 3, 24). Specifically, the level of urban economic development is gauged by the real GDP *per capita* in each city; Urban environmental pollution levels are assessed through the ratio of total waste emissions to the city’s gross urban product; Industrialization levels are determined by the proportion of secondary industry production value to the overall output of the three industries within cities; Foreign trade activity is quantified by the actual foreign investment utilized by the city; The level of public healthcare is indicated by *per capita* healthcare expenditure, specifically the ratio of local financial healthcare spending to the total population; *Per capita* education levels are represented by the average years of education per student; Urbanization levels are measured by the urbanization rate; Technological innovation is evaluated by the number of patents granted in the city; Population density is calculated as the ratio of total population to the city’s built-up area at year-end. Table 3 presents specific descriptive statistics for each variable.

**Table 3:** Statistical description of variables.

Variable	Symbols	Mean	Std.Dev	Min	Max
Digital economy	*Digi*	0.2053	0.0715	0.0511	0.5813
Health level	*Hel*	0.5452	0.1023	0.3450	0.8412
Environmental Quality	*Env*	0.8651	0.0523	0.4751	1.0125
Economic level	*Eco*	7.4780	0.7561	3.5541	11.5621
Education level	*Edu*	0.6018	0.5186	0.0341	17.9801
Industry structure	*Indu*	48.8567	9.4045	13.5723	91.2575
Health input	*Medp*	54.2413	46.3219	1.1681	386.0151
Population density	*Pden*	0.14933	0.1452	0.0013	1.5431
Foreign investment	*Fdi*	10.8274	3.4512	1.0986	13.4236
Technology Innovation	*Inno*	7.5746	1.5846	2.3026	13.8716
Urbanization rate	*City*	0.6512	0.1596	0.3381	0.9956

### 3.3 Spatial correlation analysis

Prior to initiating spatial regression analysis, it is imperative to evaluate the spatial correlation between the digital economy development level and the overall urban public health level. Typically, this examination is performed through Moran’s *I* test. The standardized spatial weight matrix for Moran’s I can be denoted as Equation 2:


(2) $$I=\frac{\sum_{i=1}^{n}\sum_{j=1}^{n}w_{ij}\left(x_{i}-\bar{x}\right)\left(x_{j}-\bar{x}\right)}{\sum_{i=1}^{n}{\left(x_{i}-\bar{x}\right)}^{2}}$$

Within the context of assessing spatial dynamics, x stands for the observed value in region i , with n representing the total number of regions. The spatial weight matrix is denoted by w . Aiming to concurrently scrutinize the influences of both distance and economic factors, this study adopts the methodology proposed by Ostherr (25). Specifically, it leverages the economic weight matrix combined with the spatial model to explore the “local-neighborhood” effects of urban digital economy advancements on public health enhancements. The findings from computing the Moran’s *I* index for both public health levels and digital economy development across cities are detailed in Table 4.

**Table 4:** Moran’s I index measurement results.

Year	Comprehensive level of health		Digital economy development level	
	Moran’*I*	*t*-value	Moran’*I*	*t*-value
2010	0.4834^***^	4.45	0.233^**^	2.17
2011	0.4237^***^	4.31	0.233^**^	2.17
2012	0.4445^***^	4.23	0.228^**^	2.18
2013	0.4132^***^	4.12	0.225^**^	2.04
2014	0.4331^***^	4.09	0.225^**^	2.08
2015	0.3251^**^	2.14	0.224^**^	2.09
2016	0.4422^***^	4.38	0.222^**^	2.07
2017	0.3831^**^	2.11	0.218^**^	2.04
2018	0.3941^***^	4.52	0.216^**^	2.07
2019	0.4151^***^	4.62	0.268^***^	9.25
2020	0.4124^***^	4.15	0.270^***^	9.17
2021	0.4125^***^	4.02	0.288^***^	8.15
2022	0.4106^***^	4.32	0.279^***^	8.29

* denotes *p* < 0.1, ** denotes *p* < 0.05, *** denotes *p* < 0.01.

The data presented in Table 4 reveals that the Moran’s *I* index for the urban public health level and digital economy development level are both positive and statistically significant. This suggests a spatial clustering effect and spatial interdependence between the two variables, warranting further examination through spatial econometric analysis.

## 4 Empirical results and analysis

### 4.1 Benchmark regression results

In the empirical investigation presented in this article, following a successful Hausman test for panel data, a panel fixed effects model was chosen for analysis. To address potential issues of structural endogeneity, the study initially employs the Systematic Generalized Method of Moments (SYS-GMM) model for regression analysis via the generalized moment estimation method, due to its capacity to more effectively handle the problems associated with weak instruments. Moreover, the results from the Likelihood Ratio (LR) test for the spatial model suggest that the spatial Durbin model outperforms both the spatial lag model and the spatial error model. Data analysis throughout this study was conducted using STATA 15.0, and the regression outcomes are detailed in Table 5.

**Table 5:** Benchmark regressions.

	Panel fixed effect		SYS	Spatial durbin	
	(1)	(2)	(3)	(4)	(5)
Digi	0.0815^***^	0.1251^***^	0.5014^***^	0.6005^***^	0.0316^***^
	(5.31)	(4.53)	(3.57)	(6.56)	(4.02)
Env		−0.1178^***^	−0.1056^***^	−0.1143^***^	−0.0076^***^
		(−4.78)	(−4.17)	(−4.61)	(−3.24)
Edu		0.0527^***^	0.5171^***^	0.4746^***^	0.0258^***^
		(7.81)	(5.80)	(4.93)	(3.16)
Indu		−0.01284^***^	−0.0291	−0.0486^***^	0.0193^**^
		(−3.74)	(−1.31)	(−4.15)	(2.05)
Eco		−0.0507	−0.0692	−0.0624	−0.0035^*^
		(−0.53)	(−0.38)	(−1.46)	(−1.75)
City		0.5675^**^	0.4201^**^	0.3215^***^	0.5231
		(2.26)	(2.33)	(2.65)	(1.36)
Medp		0.4519^**^	1.0690^***^	0.3124^***^	0.0357^**^
		(2.32)	(3.09)	(4.56)	(2.09)
Pden		0.3404	0.4493	−0.4075^***^	−0.364
		(0.51)	(1.39)	(−4.25)	(−1.51)
Inno		0.0642	0.2105	0.0521	−0.5954
		(0.67)	(1.14)	(1.52)	(−0.39)
Fdi		0.0544^*^	0.0126^**^	0.1301^**^	1.0082
		(1.75)	(2.15)	(2.36)	(1.57)
L. Hel			11.0973^***^	7.5638^***^	
			(12.79)	(9.98)	
Cons	−39.5412^***^	−4.1591^***^	−6.1986		
	(−7.25)	(−4.13)	(−1.33)		
Rho				0.1041^**^	
				(2.16)	
Hausman Test P	0.0000				
Log-like				−79.3541	
Sigma^2^				0.3213^**^	
				(2.40)	
Obe	3,420	3,420	3,135	3,135	

^***^, ^**^, and ^*^ represent 1, 5, and 10% significance levels, respectively; values in parentheses indicate *t*-values.

Regression result (1) indicates that the coefficient linking digital economy development and urban public health stands at 0.0815 without considering any other control variables, proving significant at the 1% level of significance. In contrast, regression result (2) demonstrates that with the inclusion of control variables such as urban economic indicators, education, environmental factors, and healthcare inputs, the coefficient between the two variables rises to 0.1251, still significant at the 1% level. This suggests a notably heightened impact of the digital economy on public health, with the supplementary control variables reinforcing this effect. Analysis of the control variables indicates that environmental pollution noticeably undermines urban public health enhancement (24). Conversely, increases in urban educational attainment, urbanization rates, *per capita* healthcare expenditure, and foreign investments prove advantageous for urban public health enhancement (20). Some scholars have posited that trade openness introduces healthy food options, enhances nutritional intake, facilitates technological spillovers leading to medical innovation, and ultimately benefits public health improvement (18). However, a rise in the share of secondary industries within urban areas is linked to a negative impact on public health improvement, attributed to increased emissions of wastewater, pollutants, and industrial residues that degrade environmental quality, thereby hindering public health betterment within the region (16). Interestingly, this study does not unveil a significant relationship between urban public health and urban economic development levels, population density, or the extent of technological innovation.

Given that the primary variables of interest in this study, namely the levels of urban digital economy development and urban public health, are represented by composite indicators, relying solely on panel data models for regression analysis may lead to biased estimates. To address potential endogeneity concerns, this study employs lagged explanatory variables as instrumental variables in a dynamic system generalized method of moments (SYS-GMM) analysis. The outcomes from the SYS-GMM model (Column 3) reinforce that digital economy development positively influences urban public health levels, thereby affirming, to a certain extent, the robustness of the regression findings presented in this paper.

Columns (4) and (5) of Table 5 explore the “local-neighborhood” effect of urban digital economy development on public health through the lens of the dynamic spatial Durbin model. Specifically, we investigate how the digital economy’s development in local cities impacts public health levels in both the local and neighboring cities. Column (4) addresses the impact of local digital economy development on the public health of the same city, while column (5) delves into how this local development affects the public health in neighboring cities. The regression findings suggest that digital economy advancements in local cities not only bolster the public health status within these cities but also positively influence health outcomes in adjacent areas. Further analysis of control variables reveals that environmental pollution exerts a spatial spillover effect, detrimentally impacting health across local and neighboring cities. This indicates that pollution in a city can adversely affect public health in both that city and its surrounding areas. Additionally, the spatial effects of educational human capital and medical resources have been shown to significantly enhance public health levels across both local and neighboring cities. On an industrial note, while the advancement of local industrialization appears to be detrimental to public health in the same locality, it paradoxically seems to uplift public health standards in neighboring cities. This might be attributed to local industrial agglomeration pulling industries from surrounding cities, which, while promoting industrial production locally, adversely impacts the local environment and, consequently, public health (21). Regarding regional economic growth, this study finds no significant influence on the betterment of local public health standards. Conversely, urbanization levels contribute positively to public health in local cities, with no discernible impact on neighboring regions. However, an increase in urban population density is found to be unfavorable for improving urban public health levels. Lastly, the health benefits derived from trade openness appear to be confined to the local urban contexts, highlighting the limited spatial reach of such benefits (26).

### 4.2 Robustness tests and endogeneity treatment

In our previous study, we assessed the degree of development in urban digital economy and the overall state of urban public health by utilizing an enhanced longitudinal and horizontal pull-out gearing method. To ensure the reliability of the regression outcomes, we have employed the entropy value method once more to gauge the levels of urban digital economy development and public health (21). The regression findings are detailed in columns (1) and (2) of Table 6. Even with the introduction of new explanatory variables, the analysis indicates that urban digital economy advancement continues to significantly enhance urban public health levels.

**Table 6:** Results of robustness and endogeneity tests.

	New Dig	New Hel	One period lag	Two period lag	Three period lag	Instrumental variable method	
	(1)	(2)	(3)	(4)	(5)	(6)	(7)
Digi	0.1256^***^	0.2008^***^	0.2151^***^	0.2057^***^	0.1071^***^	0.5971^*^	−0.1723^***^
	(7.58)	(4.15)	(4.53)	(4.71)	(5.57)	(1.69)	(5.41)
Control variables	Yes	Yes	Yes	Yes	Yes	Yes	Yes
Individual effects	Fixed	Fixed	Fixed	Fixed	Fixed	Fixed	Fixed
Time effect	Fixed	Fixed	Fixed	Fixed	Fixed	Fixed	Fixed
LM test	\	\	\	\	\	71.095^***^	57.269^***^
F-test	\	\	\	\	\	73.81	69.71
Obs	3,420	3,420	3,135	2,580	2,565	2,565	2,565

^***^, ^**^, and ^*^ represent 1, 5, and 10% significance levels, respectively; values in parentheses indicate *t*-values.

To investigate the dynamic impact of digital economy growth on urban public health, this study conducted further regression analysis by incorporating lagged data of urban public health levels at periods 1, 2, and 3 (25). The regression outcomes presented in columns (3)–(5) of Table 6 reaffirm the effectiveness of digital economy development in enhancing urban public health. Upon accounting for the delayed influence of the digital economy on public health levels, it was observed that the regression coefficient between digital economy development and urban public health in the lagged period is notably higher compared to the coefficient in the current period covered in the previous section. Subsequently, this coefficient gradually diminishes over time. This suggests that the efficacy of the digital economy on urban public health peaks when there is a lag of one period. Consequently, the evolution of the digital economy should undergo transformation after a specific timeframe to optimize its impact on public health. Sustained advancement in digital technology is crucial during this transition, as the effectiveness of its contribution to public health improvement may diminish otherwise (15). These results further validate the robustness of the preceding study’s conclusions.

To mitigate potential biases in regression results stemming from endogeneity issues, this study employed the *per capita* number of post offices and telephone ownership in sampled cities in 1984 as instrumental variables for measuring the digital economy. The advancement of the digital economy is intricately linked to the evolution of Internet technology. Specifically, regions with a substantial legacy of post offices and telecommunications infrastructure frequently showcase more sophisticated Internet infrastructure today (18). This selection is grounded in the understanding that the progression of the digital economy hinges on Internet technology advancement. Specifically, regions with a higher count of historical post offices and telephone sets tend to exhibit more developed Internet infrastructure today. Moreover, there exists no apparent direct link between past post office and telephone set numbers and current urban public health levels, meeting the criteria for instrumental variables concerning correlation and exogeneity conditions. Subsequent to employing the two-stage least squares method, the results of the secondary regression, displayed in columns (6) and (7), indicate that digital economy advancement continues to effectively enhance urban public health even after mitigating the endogeneity issue. These findings successfully withstand scrutiny through the LM test for non-identifiable instrumental variables and the F test for weak instrumental variables. This reaffirms the robustness and reliability of the conclusions presented in this study.

### 4.3 Heterogeneity analysis

This article delves into exploring the varying effects of the digital economy on urban public health levels, considering heterogeneity factors such as location advantages, disparities in digital economy development, economic foundations, and health inequality levels.

1. Geographic Location Influence. The regression findings in columns (1)–(3) of Table 7 reveal that regardless of whether a city is situated in the east, central, or west, the development of the digital economy can effectively enhance urban public health. Through the significance test of coefficient differences, it is found that the *p*-value of the regression coefficients for the eastern, central, and western regions was 0.063, but not significant, indicating there is no differences in the regression coefficients among the three regions. This suggests that the impact of the digital economy on improving public health is consistent across regions and aligns with the latter part of the preceding research hypothesis 3.
2. Digital Economy Development Level. By referring to the national “white paper” on smart cities and the national comprehensive pilot zone of big data, the cities studied in this paper are categorized as either smart cities—with advanced digital economies—or non-smart cities, indicating lower levels of digital economic development. Analysis from regressions (4) and (5) in Table 7 indicates that a city’s digital economy development level significantly influences urban public health, regardless of whether it is highly developed or in its nascent stages. Notably, cities with lower digital economy development show a significant regression coefficient with public health at the 10% significance level, while cities with advanced digital economies exhibit a significant impact at the 1% level. The *p*-value of the inter group coefficient difference also indicates that there is a remarkable difference in the direct regression coefficients between the two at the significance level of 5%. This pattern corresponds with the latter part of research hypothesis 4.
3. Economic Development Level. Cities are stratified into high and low economic development categories based on their average GNP. Examining regressions (6) and (7) in Table 7, it becomes apparent that the impact of digital economic development on urban public health is apparent primarily in cities with high economic development levels, whereas its effect on public health in less developed cities is not statistically significant. The *p*-value of the inter group coefficients also proves the difference between the two groups of coefficients. This discrepancy arises from the focus of less economically developed cities on advancing economic growth; only after reaching a certain economic stage can these cities allocate more resources to enhance public infrastructure like the digital economy, thereby impacting public health (15).
4. Public Health Status. Cities were distinguished based on their public health standings, categorized as either having high or low levels. Table 7 regression results (8) and (9) exhibit that in cities with lower public health standards, digital economy development significantly enhances urban public health at the 1% level of significance. Conversely, in cities with elevated public health levels, the impact of digital economy development on public health enhancement only proves significant at the 10% level. The *p*-value of the inter group coefficients also proves the difference between the two groups of coefficients. This discovery underscores that ongoing digital economy advancements, such as in the health industry and digital diagnostic and treatment technologies, effectively propel public health improvement in cities with lower health standards, while the incremental effect in cities with higher health standards is diminished, indicating a need for further advancements in digital economy development to drive continued health enhancements in these cities (11).

**Table 7:** Heterogeneous effects of the impact of the digital economy on public health.

	Region			Digital economy level		Economic development level		Health level	
	East	Central	West	Low	High	Low	High	Low	High
	(1)	(2)	(3)	(4)	(5)	(6)	(7)	(8)	(9)
Dig	0.2946^***^	0.0842^***^	0.0691^**^	0.0687^*^	0.2623^***^	0.0143	0.1085^***^	0.3239^***^	0.0153^*^
	(5.26)	(6.02)	(2.04)	(1.71)	(4.05)	(0.87)	(4.21)	(7.83)	(1.71)
Difference test *p*	0.063			0.071^**^		0.171^***^		0.025^**^	
Control variant	Yes	Yes	Yes	Yes	Yes	Yes	Yes	Yes	Yes
City FE	Fixed	Fixed	Fixed	Fixed	Fixed	Fixed	Fixed	Fixed	Fixed
Time EF	Fixed	Fixed	Fixed	Fixed	Fixed	Fixed	Fixed	Fixed	Fixed
Obs	1,212	1,200	1,008	408	3,012	420	3,000	2,028	1,392

^***^, ^**^, and ^*^ represent 1, 5, and 10% significance levels, respectively; values in parentheses indicate *t*-values. The *p* is the result of the inter group coefficient difference test conducted using the Bootstrap method to extract samples 1,000 times.

### 4.4 Impact pathway analysis

Previous mechanistic analysis reveals that the digital economy impacts urban public health levels through various mechanisms such as technological innovation, industrial upgrading, resource allocation, and the diffusion effect of information elements (1–6). To investigate the influence of digital economy development on urban public health levels, this study employs a mediating effect model. It uses the number of patent applications in cities to represent the level of technological innovation; the ratio of tertiary industry growth to depict industrial structure upgrading; total factor productivity—calculated from urban employment figures and fixed asset investments—to indicate resource allocation efficacy; and the Baidu search index for health-related searches in cities as a measure of information factor diffusion (12, 15, 16, 27).

To assess the presence or absence of mediating effects within an equation model, researchers commonly employ the stepwise regression coefficient method (28). Nevertheless, the stepwise test method exhibits low testing power, making it challenging to assess the significance of weaker mediating effects. This method may also overlook certain mediating effects that are present in practical scenarios (29, 30). In contrast, the Bootstrap method, grounded in the theoretical concept of standard error, leverages sampling with replacement to calculate an accurate standard error, assuming the sample adequately represents the population. This method, particularly when applied to large samples, offers higher statistical validity (30). Therefore, this study opts for the Bootstrap test to examine the mediating effect model. We set the Bootstrap sample size at 2000 and establish a 95% confidence interval. The outcomes of the mediation effect regression are detailed in Table 8.

**Table 8:** Regression results of Bootstrap method test for intermediate effects.

	Indirect effect coefficient	Standard error	Lower confidence limit	Upper confidence limit
Technology innovation	0.2571^***^	0.0425	0.21036	0.51368
Industrial upgrading	0.0254	0.0331	−0.17625	0.45257
Resource allocation	0.0756	0.1136	−0.12453	0.24545
Information elements	0.14523^***^	0.0354	0.21451	0.41245

^***^, ^**^, and ^*^ represent 1, 5, and 10% significance levels, respectively.

The determination of a mediating effect hinges on whether the 95% confidence interval of the indirect effect coefficient includes 0 (31). Analyzing the regression outcomes presented in Table 8, it becomes apparent that technological innovation and information dissemination significantly mediate the path of the digital economy toward enhancing public health. This finding partially validates the initial research hypotheses 1 and 4. While the digital economy’s advancement has spurred the transformation of the tertiary industry, rooted in the service sector, for further public health enhancement, progress necessitates substantial advancements in medical technology. Despite substantial governmental efforts to redress regional medical resource distribution and health disparities, urban medical resource distribution remains impacted by multifaceted factors, leading to lingering disparities in medical resource levels across Chinese cities (33). Therefore, this study’s findings diverge from prior research hypotheses 2 and 3, positing that digital economic development alone cannot enhance urban public health through industrial structure upgrades and resource optimization. This divergence highlights the nuanced complexities involved in improving public health outcomes in urban settings (13).

## 5 Main conclusions and discussion

By assessing the digital economy development and public health levels in 285 prefecture-level cities in China from 2010 to 2022, this article investigates the influence of digital economy advancement on urban public health. The main conclusions are as follows.

The advancement of the digital economy can effectively enhance the urban public health level. Even after conducting robustness treatments utilizing replacement variables and instrumental variable selection methods, this conclusion remains valid. Importantly, the positive impact of digital economy development on public health demonstrates a spatial spillover effect. This implies that improvements in a local digital economy not only boost public health within that specific area but also have positive effects on neighboring regions. Interestingly, the influence of the digital economy on urban public health is especially pronounced in cities characterized by higher levels of economic development and digital economy, alongside lower levels of public health. Technological innovation and information dissemination are the main ways for the digital economy to enhance urban public health.

Although this article investigates the relationship between the digital economy and urban public health. However, this article only analyzes the comprehensive health level of each city, without considering the differences in health levels and the degree of health inequality between cities, as well as the moderating effect of digital economy development on regional health inequality. In addition, in studying the relationship between the digital economy and urban public health, the author was limited by the difficulty of data collection and did not consider the digital literacy level of urban residents, which is their ability to utilize digital technology. These are the focus of the author’s future research.

This study proposes the following policy recommendations:

First, strengthen policies to guide the development of the digital economy: In response to the positive impact of the digital economy on public health improvement, government departments should strengthen policies to guide the development of the digital economy, encourage and support the healthy development of local digital economy, and improve the level of digital economy development. The government should enhance the modernization of social health governance and governance capabilities through digital technology innovation and digital economic governance system reform, and fully leverage the positive role of digital health. By improving regulations related to the digital economy, we aim to provide healthy digital products.

Second, promote cross departmental cooperation between the digital economy and public health: Establish a close cooperation mechanism between the digital economy and public health departments, promote information sharing and collaborative action between both parties. By applying digital technology, the quality and efficiency of public health services can be improved, ensuring the coordinated development of digital economy and public health improvement. Promote the deep integration of digital economy and health industry from policy support, technological innovation, talent cultivation, and industrial support, develop “digital economy+healthcare,” and promote digital technology to empower the health industry.

Third, strengthen public health education and publicity: Strengthen public health education and publicity work, improve residents’ health awareness and digital health literacy. According to the digital needs of different groups, improve the policies for promoting people’s health, adopt policy measures of “precise assistance,” and meet the personalized needs of different groups for digital information. Guide the public to use digital information correctly, create and regulate a digital living environment for the public, and effectively utilize digital technology to improve their own health level.

Forth, optimize the layout plan of urban digital economy: Combining the development level of digital economy and public health level, optimize the layout plan of urban digital economy, and promote the better service of digital economy to the public health field. We will focus on supporting the development of the digital health industry, improving public health services and coverage, and achieving a positive interaction between the digital economy and public health. Through equalization of digital infrastructure, we will continue to improve the fairness and accessibility of basic healthcare services, alleviate and overcome the “digital divide” between regions and urban and rural areas.

## Data availability statement

The raw data supporting the conclusions of this article will be made available by the authors, without undue reservation.

## Author contributions

HL: Data curation, Writing – original draft, Writing – review & editing. YL: Writing – original draft, Writing – review & editing.
